# Biomechanical comparison of different screw-included angles in crossing screw fixation for transverse patellar fracture in level walking: a quasi-dynamic finite element study

**DOI:** 10.1186/s13018-022-03482-x

**Published:** 2023-01-02

**Authors:** Chih-Wei Chang, Yen-Nien Chen, Hung-Chih Chang, Chun-Ting Li

**Affiliations:** 1 Department of Orthopedics, College of Medicine, National Cheng Kung University, Tainan, Taiwan; 2 Department of Orthopedics, National Cheng Kung University Hospital, College of Medicine, National Cheng Kung University, Tainan, Taiwan; 3 Department of Physical Therapy, Asia University, NO.500, Lioufeng Rd., Wufeng, Taichung, 413305 Taiwan; 4 Department of Biomedical Engineering, Hungkuang University, Taichung, Taiwan; 5 Institute of Geriatric Welfare Technology & Science, MacKay Medical College, No.46, Sec. 3, Zhongzheng Rd., Sanzhi Dist., New Taipei City, 25245 Taiwan

**Keywords:** Patellar fracture, Crossing screw fixation, Parallel screw fixation, Quasi-dynamic simulation, Level walking

## Abstract

**Background:**

A minimally invasive technique with various screw configurations without open surgery is currently used for the fixation of transverse patellar fractures. Percutaneous crossing screw configuration has been reported to have a good bone union rate in patellar fractures. However, the difference in mechanical stability of the fractured patella between different screw-included angles has not been fully investigated. Hence, this study aims to compare the mechanical stability of parallel and crossing screw fixations with different screw-included angles for the fixation of transverse patellar fractures during level walking.

**Methods:**

A finite element knee model containing a patella with a transverse fracture is created. Two headless compression screws with different angles (0°, 30°, 60°, and 90°) are used to fix the fracture. The loading conditions of the knee joint during level walking are used to compare the stability of the fractured patella with different fixation screw configurations.

**Results:**

The results indicate that the maximum fracture gap opening distance increased with an increase in the included angle. Two parallel screws yield the smallest gap distance among all screw configurations. The maximum gap opening distances at the anterior leading edge of the fractured patella with two parallel screws and two screws having an included angle of 90° are 0.73 mm and 1.31 mm, respectively, at 15% walking cycle.

**Conclusions:**

Based on these results, the superior performance of two parallel screws over crossing screw fixations in the fixation of transverse patellar fractures is established. Furthermore, the smaller the angle between the crossing screws, the better is the stability of the fractured patella.

## Introduction

The patella, embedded in the patellar tendon, plays an important role in transferring the quadriceps force to the tibia to develop the extensor mechanism of the knee joint [1]. The extensor mechanism of the knee joint is essential for normal walking and daily activity. When the patella is fractured, the extensor mechanism is interrupted and knee movement is blocked. Unfortunately, patellar fracture is a common disorder that orthopedic surgeons encounter in clinical examination, particularly in the emergency room [2, 3]. Surgical intervention is suggested for cases of displaced patellar fractures to firmly fix the patellar fragments and restore the knee extensor mechanism to enable walking [4, 5]. In addition, surgical intervention allows early activity to avoid complications due to long-term immobilization of the knee joint.

To date, several surgical approaches have been proposed for the management of patellar fractures, including tension band wiring, cerclage, modified tension bands, pins or screws, and combined approaches [6–10]. According to the literature, more than half of those cases with patellar fracture accepted tension band wiring from 2003 to 2015, while the combined approach has increased recently [11]. The major advantage of the combined approach, such as cannulated screws along with an anterior wire, is its excellent stability [12, 13]. Cannulated screws along with an anterior wire were also reported to be superior to tension band wiring in the literature [14]. However, the major disadvantage of cannulated screws with an anterior wire is delayed healing and pain after surgery due to damage to the soft tissue because of open surgery [15–17]. Hence, several studies have focused on minimally invasive surgery without the anterior wire and with a specific cable-pin system, two and three parallel headless compression screws, and full-thread screws to enhance the healing process and reduce pain [12, 13, 18, 19].

A percutaneous crossing screw configuration without an anterior wire has also been proposed to achieve a good union rate for patellar fractures [20]. However, the difference in mechanical stability of the fractured patella between the two parallel screws and two crossing screws during daily walking is unclear. Hence, the aim of this study is to compare the stability of the fractured patella with different screw-included angles in the fixation of transverse patellar fractures in level walking by using finite element (FE) simulation. The reason for using FE simulation is the strength in solving such a highly nonlinear lading of the patella during walking. FE modeling has been used in many biomechanical studies, particularly with complex geometries and loadings [21].

## Method

### Solid modeling

**Fig. 1:**
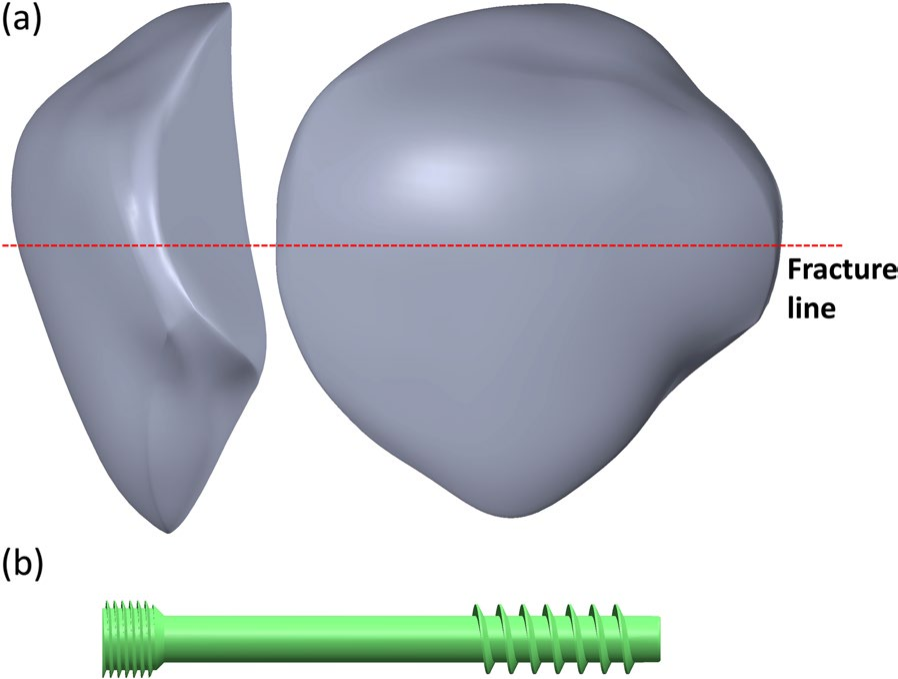
Patella (**a**) and headless compression screw (**b**) used in the simulation

A knee joint model containing the patella, distal femur, proximal tibia, and fibula was developed based on the computer tomographic images of a healthy man with a body weight of 70 kg and height of 170 cm. The images were obtained when the subject was lying supine; hence, the knee model was created in the full extension position. The areas of the cortical and cancellous bone in the images were isolated out by the higher gray values than the surrounding soft tissues. The 3D model of the bones was then developed by stacking the isolated bony areas. It was subsequently imported into the CAD software, SolidWorks 2019, to create the cartilage and meniscus. The thickness of the cartilage of the patella, distal femoral, and proximal tibia was set at 1 mm based on the literature [22]. The space between the cartilage was created as a meniscus. The transverse fracture type AO 34-C1 at the middle of the patella was used in this study (Fig. 1a). The transverse fracture was created by a virtual plane; hence, no gaps existed after the section. After the fracture was created, a 4.5 mm headless compression screw (HCS, DePuy Synthes, Raynham, MA, US) was used to fix the fractured patellar fragments (Fig. 1b).

**Fig. 2:**
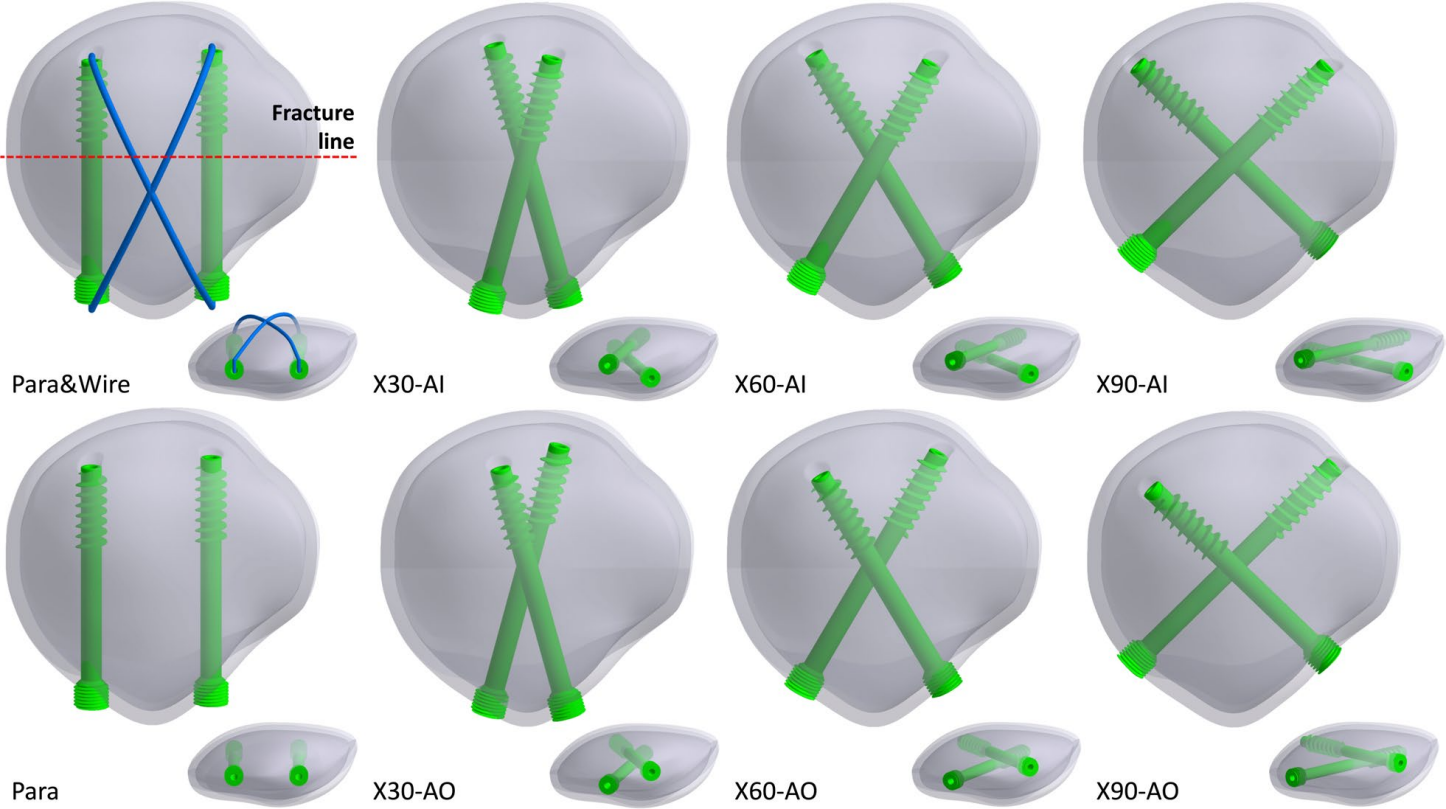
Screw configurations used in the present study

To investigate the effect of the included angle of the screws in crossing fixations, three crossing angles were considered: 30°, 60°, and 90°. Furthermore, in the crossing screws, there are two different screw configurations: the anterior screw inward and anterior screw outward. In addition to cross-fixation, traditional parallel screw fixation was used for comparison. Two parallel screws were placed in the middle third of the patella in the front plane. The proximity of the screws was approximately half of the thickness of the patella. Additionally, the currently used anterior wire in a figure of eight with two parallel screws was used for validation. The diameter of the anterior wire was set at 1 mm. The length of the screws ranged from 35 to 40 mm, and the thread length was 12 mm. The thread was controlled to avoid passing through the fracture site; hence, compression and separation of the fractured patellar fragments under loading were allowed. In total, eight different configurations were used (Fig. 2): two parallel screws (Para), two parallel screws with anterior wire (Para&Wire), crossing 30°with the anterior screw inward (X30-AI), crossing 30°with the anterior screw outward (X30-AO), crossing 60°with the anterior screw inward (X60-AI), crossing 60°with the anterior screw outward (X60-AO), crossing 90°with the anterior screw inward (X90-AI), and crossing 90°with the anterior screw outward (X90-AO).

### FE modeling

The model was imported into ANSYS Workbench 2022 for simulation. A quadratic tetrahedron element (solid 187) was used to mesh the complex model, including the bone, cartilage, screw, and wire. The ligaments of the knee joint were reconstructed using tension-only springs in the ANSYS Workbench. In total, five ligaments (Table 1), namely the patellar, medial collateral, lateral collateral, anterior cruciate, and posterior cruciate ligaments, were created in the FE model. The stiffness of the spring was defined based on literatures [23–26]. The material properties of the bone, cartilage, and meniscus were set according to literature (Table 2) [27–31]. The material properties of the metal were used based on the engineering database in the ANSYS Workbench. All the materials were as assumed to be linear elastic, isotropic, and homogeneous.

**Table 1:** The stiffness of the springs used in this study

	Stiffness of single spring (N/mm)	Spring Numbers	References
Patellar ligament	24	44	[26]
Medial collateral ligament	25	3	[25]
Lateral collateral ligament	24	3	[25]
Anterior cruciate ligament	120	2	[24]
Posterior cruciate ligament	120	2	[23]

**Table 2:** The material properties used in this study

	Elastic modulus (MP)	Poisson’s ratio	References
Femur, tibia and fibula			
Cortical bone	17 500	0.3	[27, 28]
Cancellous bone	600	0.3	[27, 28]
Patella			
Cortical bone	1 000	0.3	[29]
Cancellous bone	207	0.3	[29]
Articular cartilage	100	0.3	[30]
Meniscus	300	0.3	[31]

### Model validation and convergence test

A convergence test was conducted with the Para model in the full extension position to confirm that the mesh status of the FE model was stable. The total number of elements was increased by globally reducing the edge length of the element to confirm the stability of the numerical model. The maximum gap distance between the fractured patella and contact area between the patellar fragments were used as indices. After convergence, to validate the present FE model, the rotational degree in the sagittal plane of the patellar fragment Para&Wire model was compared to the proposed result in a cadaveric study [32].

### Walking simulation

**Fig. 3:**
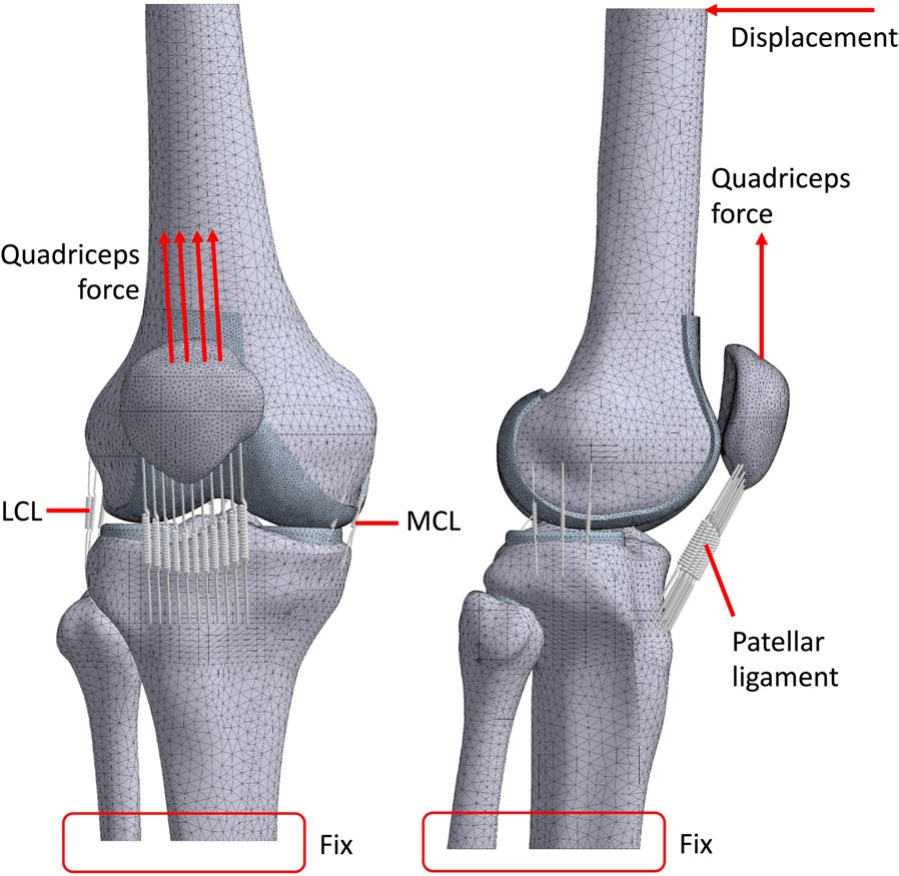
Finite element model and boundary conditions for walking simulation

After validation and convergence, the motion of the knee joint during level walking was simulated by applying rotational degrees to the femur. Normalized displacement was applied to the superior surface of the femur, while the distal end of the tibia and fibula were completely fixed (Fig. 3). In total, 20 positions with a sequential increment of 5% in the walking cycle were used (Table 3). All internals were consistent, and the endpoint of the interval was the starting point of the following interval. Linear interpolation was used for each interval. In addition to the displacement, a quadriceps force parallel to the long axis of the femur was applied to the base surface of the fractured patella. The applied degree and force data were set based on a previous study [33]. During the late swing phase, quadriceps force was set to 1 N for solving.

**Table 3:** The used gait data in this study

Stance (% gait cycle)	5	10	15	20	25	30	35	40	45	50
Knee flexion (Degree)	7	14	18.6	15.1	14	10.5	8.1	5.8	5.8	8.1
Quadriceps (N)	136	272	836	645	318	127	27.3	27.3	27.3	27.3
Stance (% gait cycle)	55	60	65	70	75	80	85	90	95	100
Knee flexion (Degree)	15.1	27.9	43	57	60.5	55.8	43	24	3.5	0
Quadriceps (N)	127.3	254.5	254.5	136.4	36.4	18.2	1	1	1	1

### Index

The gap opening distances at the anterior, medial, and lateral sides of the fracture gap during walking were used as indices for comparison. Furthermore, the change in the contact area between the proximal and distal patellar fragments with different configurations during walking was plotted for comparison.

## Results

### Convergence and validation

**Fig. 4:**
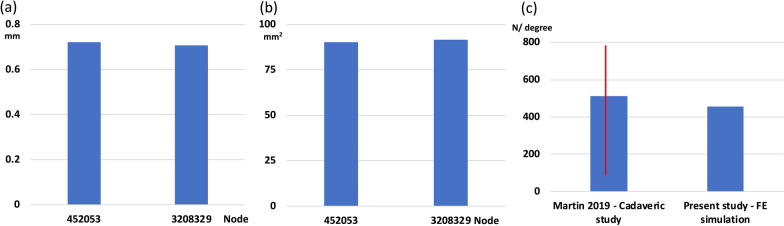
Comparison of gap opening distance (**a**) and contact area (**b**) in the convergence test, and rotational stiffness in the validation (**c**)

The difference in maximum gap opening distance at the anterior gap was 1.4% (from 0.72 to 0.71 mm) between 452 053 and 3 208 329 nodes. Additionally, the difference in the contact area between the patellar fragments was 1.4% (from 90.1 to 91.4 mm^2^) between 452 053 and 3 208 329 nodes (Fig. 4a and b). Furthermore, the rotational stiffness of the Para&Wire model was similar to that in a previous study [32]. The results of the rotational stiffness in the present and published studies were 453 N/degree and 510 (SD 362) N/degree, respectively (Fig. 4c).

### Gap distance

**Fig. 5:**
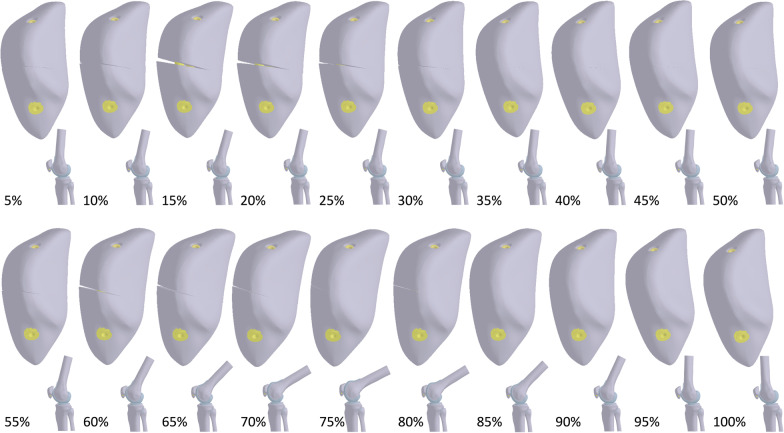
Movement of the knee joint in the simulation

**Fig. 6:**
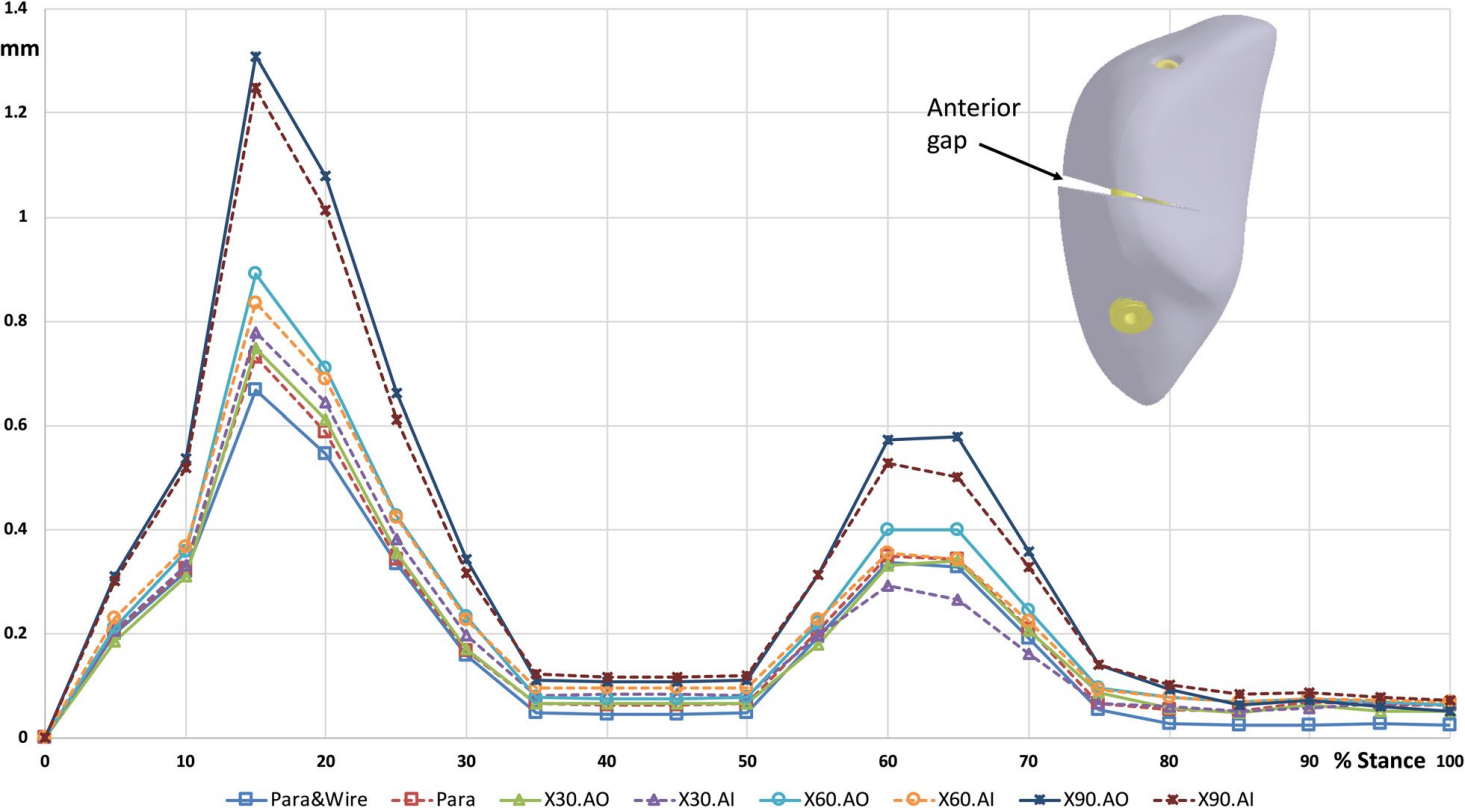
Anterior gap opening distance during walking cycle

**Fig. 7:**
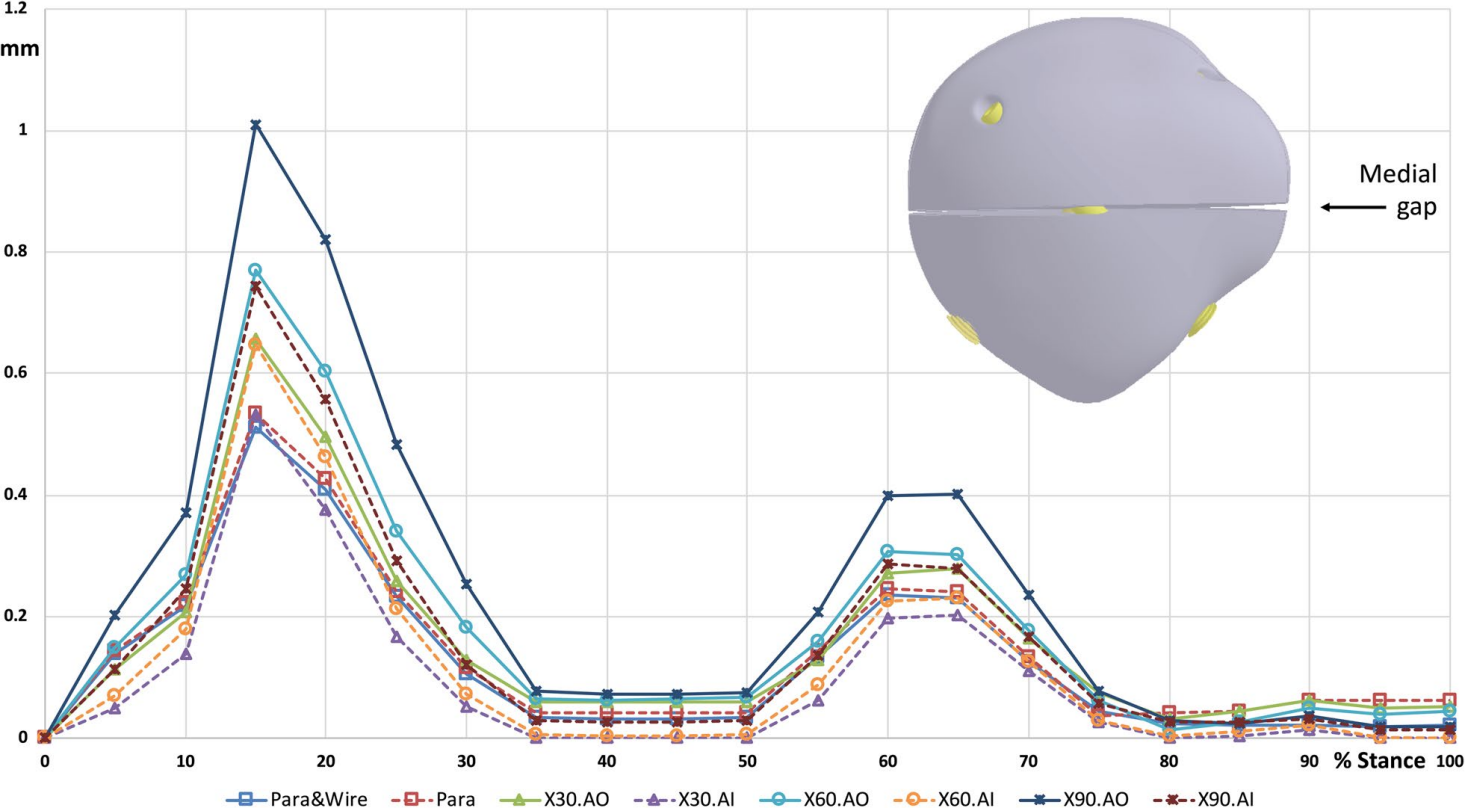
Medial gap opening distance during walking cycle

**Fig. 8:**
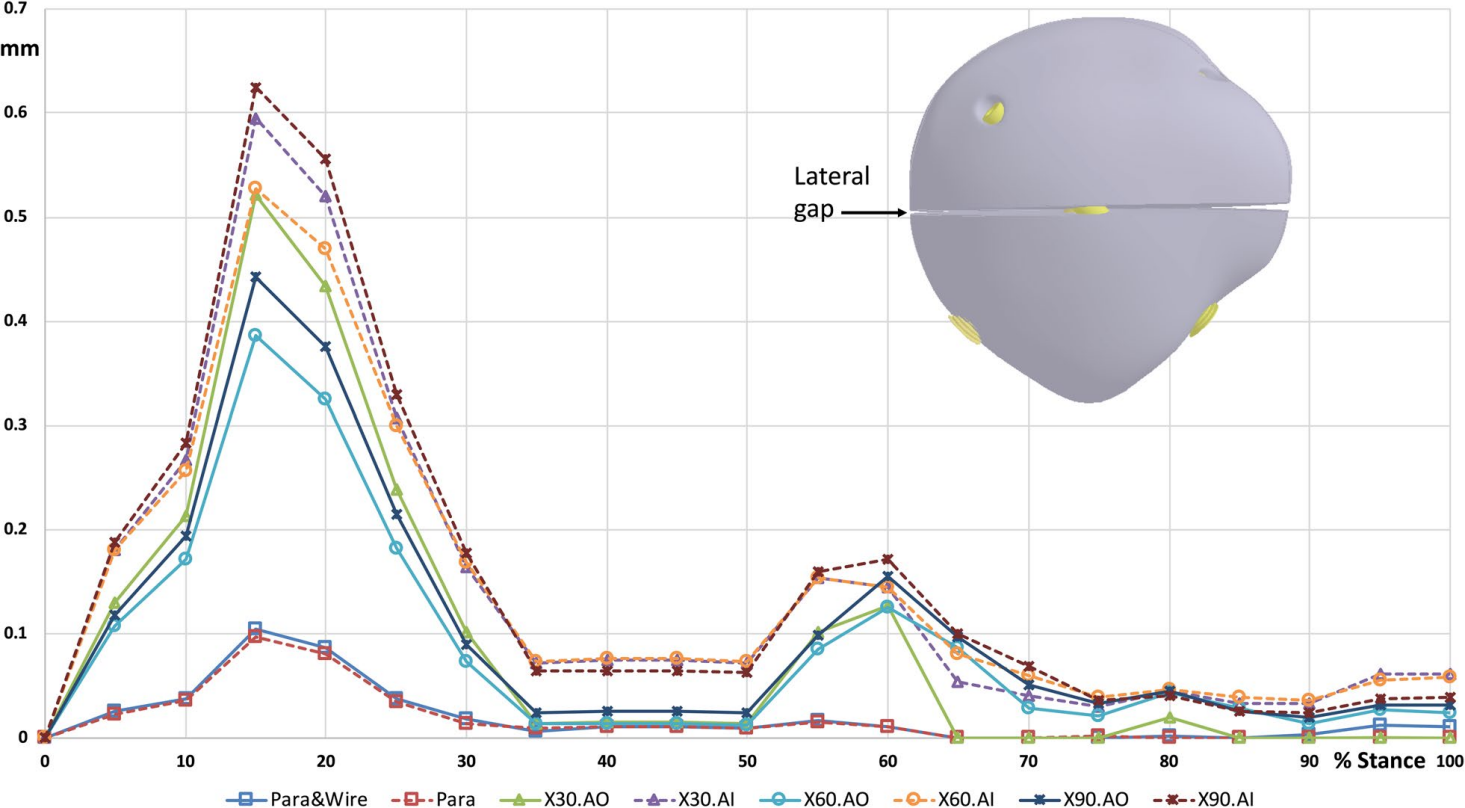
Lateral gap opening distance during walking cycle

The fractured patella separated at the anterior surface, and a large gap was developed, while the posterior aspect remained in contact with each other during walking (Fig. 5). Parallel screw fixation, regardless of whether the anterior wire was used, yielded a smaller gap opening distance than crossing screw fixation. Furthermore, the maximum gap opening distance during walking increased with an increase in the included angle in the crossed screws. In stance phase, the maximum gap opening distance of X90.AO was 1.25 (Fig. 6), 1.01 (Fig. 7) and 0.63 mm (Fig. 8) at anterior, medial, and lateral site of the gap, respectively, at 15% of the walking cycle. In swing phase, the maximum gap opening distance of X90.AO was 0.57, 0.4 and 0.16 mm at the anterior, medial, and lateral site of the gap, respectively, at 60% of the walking cycle. In general, the anterior gap opening distance was larger than the medial and lateral gaps during the walking cycle.

### Contact area

**Fig. 9:**
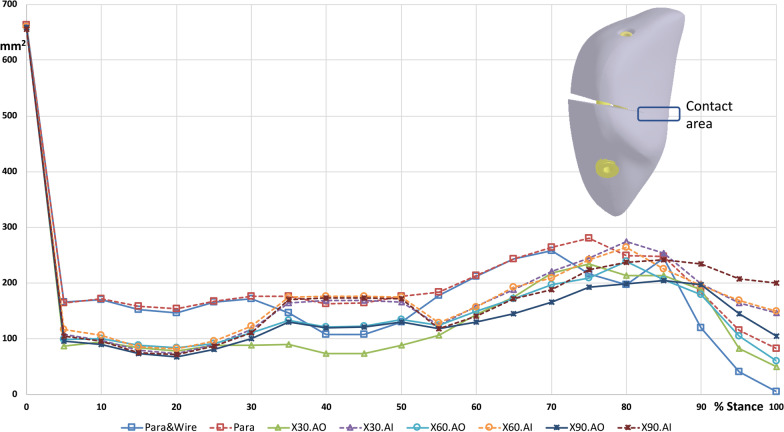
Contact area between the fragments during walking cycle

The contact area of the patellar fragments at the fracture site with parallel screw fixations was obviously larger than that with crossing screw fixations in the stance phase (5–30% of the walking cycle) (Fig. 9). The contact areas in the fracture patella with different fixations were similar during the swing phase. The maximum contact areas in the Para model were 176 mm^2^ and 280 mm^2^ in the stance phase and swing phase, respectively. The difference in contact area between parallel screw fixation with and without anterior fixation was very minor.

## Discussion

This novel study demonstrates loading on a fractured patella with different screw configurations in level walking. The present simulation could demonstrate the difference in fracture gap deformation between the stance and swing phases of the walking cycle. The loading conditions of the patella differ during walking, and the direction of the muscle force changes during knee flexion and extension. In most previous studies, a worst-case loading condition of the fractured patella was assumed, and the gap opening distance was demonstrated at specific knee flexion angles [12, 32, 34]. In this simulation, the loading condition was extended to the entire walking cycle. Determining the gap opening of the fractured patella with different screw fixations is helpful to surgeons and physical therapists in the decision-making process of postoperative care.

Patellar fracture is a common fracture encountered by orthopedic surgeons in clinical practice. The treatment strategy for fractures is still evolving because new devices and surgical approaches are continuously being proposed. In recent years, the conventional approach, that is, tension band wiring, has gradually decreased, while the combined approach has become popular [11]. Some specific systems with minimally invasive techniques, such as the cable-pin system, have been proposed to reduce post-surgery pain compared with conventional approaches [18]. In addition, many approaches with percutaneous screw fixation without the anterior wire, such as two parallel headless compression screws, two parallel full-thread screws, crossing screws, and triangular screw configurations, have been proposed recently [12, 13, 20, 35]. All approaches satisfy the criteria of a minimally invasive technique.

Crossing screw fixation with a minimally invasive technique for transverse patellar fractures was proposed to achieve a good union rate in previous studies [20]. In a previous study, the two screws were placed nearly perpendicular to each other during fixation. In the present study, three different screw-included angles were employed, and the effect of the screw angle on the stability of the fractured patella was compared. The results indicated that the gap opening distance increased with an increase in the included angle. The major loading on the fractured patella comes from the quadriceps force and the distal femoral condyle, which develops a torque on the sagittal plane. The anterior leading surface of the fracture opened under such torque. Under such loading conditions, the screw location was closer to the anterior surface of the patella, and higher stability was obtained. One of the screws in the crossing fixation was deeper than that in the parallel screw fixation; hence, the stability with crossing fixations was lower than that with parallel screw fixation.

In the present simulation, the results indicated that the stability of the fractured patella with crossed screw fixation was lower than that with parallel screw fixation. However, crossed screw fixation with an included angle of 90 °was reported to achieve good clinical outcomes [20]. Mechanical stability is not the only factor affecting fracture healing. A minimally invasive approach that can provide sufficient blood supply with preservation of the periosteum and stabilization of the fracture is one of the options usually considered by the surgeon in fracture fixation [18, 35–37]. Percutaneous crossing screw fixation is performed using a minimally invasive technique without open surgery; hence, the outcome of bone healing is favorable. Additionally, good postoperative care, including protection of the fractured bone, remains indispensable.

The use of an anterior wire has been shown to achieve higher stability than that without it [19, 38]. However, the most critical aspect of the anterior region is the damage to the soft tissue surrounding the fractured patella [16]. Many studies have indicated lesser pain after surgery in cases without the anterior wire than in cases without the anterior wire [18]. In our previous studies, the effect of the anterior wire on the stability of the fractured patella was not obvious when the screw was superficially placed [19]. In the present study, screw proximity was similar to that in the previous study, and more complex loading conditions were applied to the fractured patella with and without the anterior wire. However, the differences in the gap opening distance and contact area in the walking cycle with and without the anterior wire were still very minor. This indicates that the effect of the anterior wire on the stability of the fractured patella was related to screw proximity, regardless of the flexion degree of the knee joint.

The present quasi-dynamic simulation has several limitations. First, inertial force was not considered in the simulation. Second, the motion and force data for the boundary conditions were not directly measured from the subject who accepted the CT scan and provided CT images. Third, only the quadriceps force was considered, and the muscle forces from the hamstring and gastrocnemius were not considered. Fourth, the ligaments around the knee joint were simplified as a spring, and all the materials were simplified as linear elastic, isotropic, and homogeneous.

## Conclusion

This novel FE simulation of knee model can represent the mechanical responses of a fractured patella with different screw fixations during level walking, and the fracture gap opening distance and contact area between fragments are demonstrated. Based on these results, the two parallel screws are noticed to have superior performance than the crossing screw fixations in the management of transverse patellar fractures with headless compression screws. Furthermore, the smaller the angle between the crossing screws, the better the stability of the fractured patella when two headless compression screws are used.

## Data Availability

All the data will be available upon motivated request to the corresponding author of the present paper.

## Abbreviations

FE: Finite element
Para: Two parallel screws
Para&Wire: Two parallel screws with anterior wire
X30-AI: Crossing 30° with the anterior screw inward
X30-AO: Crossing 30° with the anterior screw outward
X60-AI: Crossing 60° with the anterior screw inward
X60-AO: Crossing 60° with the anterior screw outward
X90-AI: Crossing 90° with the anterior screw inward
X90-AO: Crossing 90° with the anterior screw outward
